# Electroporation Assisted Improvement of Freezing Tolerance in Yeast Cells

**DOI:** 10.3390/foods10010170

**Published:** 2021-01-15

**Authors:** Povilas Simonis, Ausra Linkeviciute, Arunas Stirke

**Affiliations:** Center for Physical Sciences and Technology, State Research Institute, Saulėtekio al. 3, T-02300 Vilnius, Lithuania; ausra.linkeviciute@ftmc.lt (A.L.); arunas.stirke@ftmc.lt (A.S.)

**Keywords:** yeast, cryoprotection, trehalose, electroporation

## Abstract

Prolonged storage of frozen dough worsens the structure of thawed dough. The main reason is the inhibition of yeast activity. In this study we investigated applicability of pulsed electric field treatment for introduction of cryoprotectant into yeast cells. We showed that pre-treatment of cells suspended in a trehalose solution improves freezing tolerance and results in higher viability after thawing. Viability increased with rise in electric field strength (from 3 to 4.5 kV/cm) and incubation time (from 0 to 60 min) after exposure. Pretreatment resulted in lower decrease in the viability of thawed cells, viability of untreated cells dropped to 10%, while pre-treatment with PEF and trehalose tripled the viability.

## 1. Introduction

The development of sustainable solutions for food waste management represents one of the main challenges for society. These resolutions should be capable of exploiting the resources represented by food waste to achieve social, economical and environmental benefits. One of the directions that could improve food waste management is the development of new freezing technologies [1]. Freezing is an advantageous processing method that increases the shelf-life of many products including yeast-leavened doughs [2]. The main disadvantage of such a preservation technique is that texture and loaf volume gradually deteriorates. The decrease of yeast viability and gluten network loses by recrystallization of ice crystals contributes to the quality loss of yeast-leavened dough as well [3].

Preservation of yeast cell viability is important in both research and the food industry. In research the viability of cells is required to maintain strains possessing useful traits for long periods of time. Meanwhile, in the food industry the viability of yeast cells is required for efficient fermentation of food products like frozen dough [4]. In both fields of application preservation is often achieved by subjecting yeast cells to low temperatures. Refrigeration leads to the formation of ice crystals and the removal of water from within the cells [5]. Freezing and thawing subsequently can cause damage on cell wall, membranes, induce oxidative stress leading to potential gene mutations and decreased cell survival. After thawing cell stocks or dough, yeast cells show dramatically decreased viability followed by lower fermentation activity [6].

Freezing injuries in yeast cells depend on various factors including the physiological state and freezing conditions [7]. If the cooling rate is slow, intracellular water will have time to flow out by osmosis, and ice crystals will primarily form extracellularly. Water removal from the intracellular space leads to shrinkage of cells and the concentration of solutes (potentially toxic). During fast cooling intracellular water has no time to flow out and thus ice crystals will form intracellularly [8]. Therefore, optimal conditions for a specific cell type should be chosen.

Data from analyzing various cold-tolerant organisms shows that quite different compounds could provide protection from cryodamage. These cryoprotectants inhibit crystal growth, help to retain intracellular water and stabilize intracellular compounds [9]. In yeast cells such naturally occurring protective compound is trehalose. Multiple studies show that tolerance to freezing is strongly related to the concentration of intracellular trehalose [10,11,12]. The concentration of a cryoprotectant can be increased by manipulating the genetic background [13], but such measures can cause long term perturbations in the overall metabolism. Besides bioavailability, the localization of a cryoprotectant is of major importance as well. Cryoprotectants have benefits both extracellularly and intracellularly [10], but only some cryoprotectants could enter the cells straight from the media. The main limiting factors are size, charge and a lack of active transport systems. Under regular conditions, the content of intracellularly occurring cryoprotectants is defined by the metabolism of specific cells and is limited to only a small number of cryoprotectants. One of the techniques that could widen the range of applicable cryoprotectants by weakening membrane barriers is exposure to pulsed electric fields (PEF). It is a process that can induce the formation of pores within plasma membrane and result in higher permeability. 

The goal of this study is to investigate whether exposure to PEF could be used for fast, efficient introduction of cryoprotectants into yeast cells and increase their tolerance to freezing.

## 2. Materials and Methods 

### 2.1. Materials 

*S. cerevisiae* BY4742 (*MAT*α; *his*3∆1; *leu*2∆0; lys2∆0 and *ura*3∆0) yeast cells were purchased from Euroscarf (Oberursel, Germany). Yeast extract was obtained from BioCORP (Warsaw, Poland). Peptone ex casein was purchased from Carl Roth GmbH (Karlsruhe, Germany). Agar was obtained from Alfa Aesar (Haverhill, US). Sorbitol, TRIS and D (+)-Trehalose dihydrate (CAS-No. 6138-23-4) as a trehalose standard were purchased from AppliChem GmbH (Darmstadt, Germany). Glucose and HCl were obtained from Merck KGaA (Darmstadt, Germany). Formic acid and acetonitrile were of high performance liquid chromatography (HPLC) grade and obtained from Sigma-Aldrich Chemie GmbH (Steinhem, Germany) and ethanol (99.5%) from Merck KGaA (Darmstadt, Germany). The water used in this study was cleaned via the Millipore Direct Q water purification system (Merck KGaA, Darmstadt, Germany).

### 2.2. Cultivation and Manipulation of Yeast Cells 

Yeast cells were grown in liquid media YPD (1% yeast extract, 2% peptone ex casein and 2% glucose) at 30 °C in a reciprocal shaker operating at 200 rpm. Cell concentration was measured by optical density (OD) using an absorption spectrophotometer (Halo RB-10, Newport Pagnell, Dynamica Scientific Ltd., Livingston, UK) at 600 nm wavelength. Yeast cells were harvested after reaching 0.8–1.2 OD (early exponential phase). Cells were then washed twice with electroporation buffer (20 mmol/L TRIS, HCl, pH 7.4), and resuspended in electroporation buffer supplemented with 1 M D (+)-Trehalose dihydrate (EPBT) or 1 M sorbitol (EPBS). Before exposure to electric fields yeast cell suspensions were soaked on ice for 15 min.

Freezing was performed by transferring 100 µL of cell suspension into plastic PCR tube and placing it into freezing chamber at −20 °C. Frozen cells were kept for 4–40 days. Thawing was performed in a water bath at 20 °C.

The viability was evaluated by plating treated cell suspension onto a solid media (YPD with 1.2% agar) and then incubating at 30 °C for 48 h. After incubation, the number of colony-forming units (CFU) was evaluated. As a control, PEF untreated suspension was used.

Significance of obtained data was evaluated by the Tukey–Kramer multiple comparison procedure [14].

### 2.3. Extraction of Intracellular Trehalose and Its Preparation for the Detection and Quantification

After soaking in EPBT yeast cells were collected by centrifugation. In order to remove the extracellular trehalose, the pellet was washed with EPBS and concentrated again by centrifugation. Supernatant was completely removed and pellet was suspended into boiling ethanol. Extraction was performed by disintegrating cells for 15 min in ultrasound bath at 75 °C. The pellet was concentrated by centrifugation for 10 min at 13,000 rpm. Of the supernatant 1 mL was collected and evaporated to dryness under a stream of nitrogen gas. The dry residue was dissolved in 1 mL of acetonitrile and water solution (50:50, *v*/*v*) and analyzed via liquid chromatography–mass spectrometry (LC–MS).

### 2.4. LC–MS Analysis

The LC–MS assay was prepared according to the method of Zhiqian and Simone Rochfort [15] with some modifications. The LC separation was achieved using an Agilent Zorbax RX–SIL (5 µm, 250 × 4.6 mm) column (Agilent Technologies, Santa Clara, CA, USA) coupled with Zorbax RX–SIL (5 µm, 12.5 × 4.6 mm) guard column (Agilent Technologies, Santa Clara, CA, USA) on an Agilent 1260 Infinity Liquid Chromatography system (Agilent Technologies, Waldbronn, Germany) equipped with a quaternary pump, a vacuum degasser module, an autosampler, and a column compartment (maintained at 30 °C). Separations were carried out in the binary solvent system: solvent A, 0.1% formic acid in water and solvent B, 0.1% formic acid in acetonitrile. The flow rate was 0.75 mL/min with an isocratic elution of 75% solvent B over 15 min. The injection volume was 10 μL. Analyte detection was by an Agilent 6224 Accurate-Mass Time-of-Flight mass spectrometer (Agilent Technologies, Santa Clara, CA, USA) with an electrospray ionization (Dual-ESI) source. The mass spectrometer was operated with full scan (50–800 m/z) in the negative ion trap mode for sugar analysis. The MS conditions are listed in Table A1 (see in Appendix A).

The m/z value of 387 corresponding to formate adduct ion [M+HCOO]- for trehalose was extracted from the full scan chromatograms (i.e., TIC→EIC) and peak areas were integrated using an Agilent MassHunter Quantitative Analysis software (Agilent Technologies, Santa Clara, CA, USA).

Determination of the limit of detection (LOD) and limit of quantitation (LOQ) and the linear range for trehalose was carried out using a series of seven dilutions (ranging from 0.0001 to 0.1 mg/mL). Calibration curves were generated in the software by plotting the relative response to trehalose concentration. Averages of the responses for each standard were calculated and plotted against the known concentrations. These plots were used for any further calculations when determining the LOD and LOQ, calculating the trehalose concentration of samples (Table 1).

### 2.5. PEF Generation System

A pulse generator assembled in the Center for Physical Sciences and Technology was used in the experiments [16]. 400 µL of the yeast cell suspension was placed into a cuvette with 2 mm gap between aluminum electrodes (BTX, UK) and exposed to a single square-shaped pulse with a pulse length of 150 µs and an electric field strength (E) of up to 8 kV/cm.

## 3. Results and Discussion

**Figure 1:**
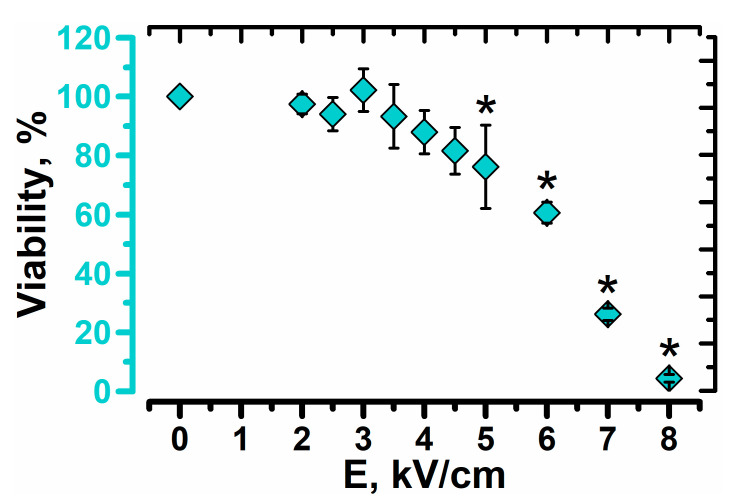
Viability of yeast cells after exposure to single electric field pulse of different field strengths (E) and duration of 150 µs. Asterisks (*) indicate significant difference (*p* < 0.05) in the viability when compared to PEF-untreated cells.

Investigation of electric field induced effects was performed by analyzing yeast cell viability (Figure 1). After exposure to the electric field pulse yeast cell suspension was kept on ice for 30 min, diluted and plated on growth media. In order to find optimal treatment conditions for the introduction of cryoprotectant, cell suspension was exposed to pulses with different electric field strengths (E ≤ 8 kV/cm).

We showed that the viability of yeast cells starts to decrease when an electric field strength of a pulse exceeds 4 kV/cm. This value indicates a threshold above which some cells experience irreversible damage to their components and in turn leads to cellular death. In order to investigate this threshold in more detail, improvement of freezing tolerance will be performed by exposing yeast cells to PEF when E is between 3 and 5 kV/cm. Such pulses are likely to increase the permeability of plasma membrane with a minor impact on the viability. Some lethal effects of pulsed electric field on yeast cells, like irreversible permeabilization or activation of metacaspases, were investigated and discussed previously [17,18,19,20].

It is important to remark that during PEF treatment procedure, trehalose acts not only as a cryoprotectant, but also as an osmoregulator. It was previously calculated that isotonic intracellular osmolality of yeast cell protoplasts is approximately 0.54 Osm [21]. Transferring cells into hypertonic solution of 1 Osm can result in a decrease of cell volume by up to 40% [22]. In this research we used a solution with osmolality close to 1 Osm, which after transferring cells from growth media could cause mild osmotic shock and decrease in the volume of cells. To allow some adaptation, yeast cells were soaked for at least 15 min before further treatment. Such preparation was constant in the scope of this study. Therefore, the correlation between soaking in trehalose and PEF treatment was not evaluated. Since the extent of electroporation induced damage depends on the diameter of a cell [23], the decrease in volume could possibly contribute to weaker permeabilization. Higher than isotonic osmolality is also used as a preventative measure against excessive leakage of intracellular compounds after exposure to pulsed electric fields [24].

Another important aspect regarding the viability of yeast cells after exposure to PEF is the soaking temperature. Before and after exposure yeast cells were kept on ice. It was previously shown that keeping mammalian cells on ice prior to transfection increases its efficiency [25]. Since membrane resealing kinetics depend on the temperature [26], we assume that keeping cells on ice increase membrane resealing time thus allowing absorption of more cryoprotectant. Other researchers have shown that soaking for 3 h at 10 °C in 10% trehalose solution prior to freezing can have significant impact on freezing tolerance in yeast cells [27]. Yet our goal was to reduce soaking time needed for freezing tolerance improvement.

**Figure 2:**
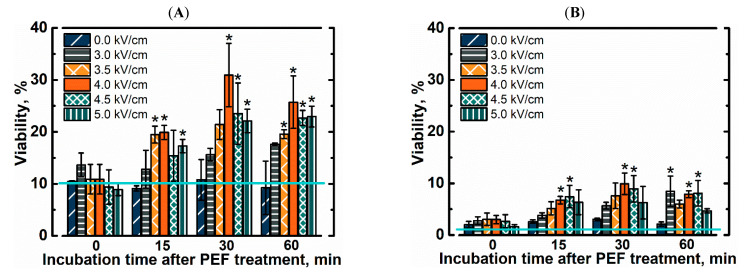
Viability of yeast cells after exposure to single electric field pulse and subsequent freezing for (**A**) 4 days or (**B**) 40 days at −20 °C. Teal line represents viability of yeast cells without soaking in trehalose and pulsed electric field (PEF) treatment. Asterisks (*) indicate significant difference (*p* < 0.05) in the viability when compared to PEF-untreated cells with the same soaking time.

In order to evaluate freezing effects on yeast cells we kept yeast cell suspension at −20 °C from 4 to 40 days. Viability after freezing, with and without PEF pretreatment is shown in Figure 2. In this research we used yeast cells from the early exponential growth phase, which contain only minimal amounts of intracellular trehalose [28]. Even after 4 days of freezing, the viability of untreated yeast cells dropped to 12%. It was previously shown that yeast cells from the exponential growth phase are more sensitive to freezing than the ones from the stationary growth phase [8]. In this study soaking of yeast cells in 1 M trehalose solution for up to 75 min did not result in significant improvement in the viability after freezing. Results imply that such duration is too short to import or synthesize significant amounts of trehalose under our experimental conditions. It was previously shown that long term soaking of yeast cells in 1 M trehalose solution results in higher resistance to freezing without significant decrease in leavening ability [29]. The downside of simple soaking is that it can take days to see significant changes and cause further aging of cells. In order to decrease the length of the pretreatment procedure we tested exposure to PEF as a method to introduce trehalose.

The yeast cell suspension was exposed to a single electric field pulse and thereafter soaked in the same solution for up to 60 min before freezing. We show that the viability of pretreated yeast cells depends on a soaking time after PEF pretreatment. No significant improvement in freezing tolerance was observed if cell suspensions were transferred in to the freezer immediately after exposure to the electric field pulse. The viability of thawed yeast cells increased with a rise in soaking time and was higher by up to 3 times when compared to the viability of PEF untreated cells. The strength of an electric field pulse was an important parameter as well. Viability of pretreated cells after thawing increased with a rise in strength of a pulse (up to 4 kV/cm). Stronger electric fields still improved freezing tolerance when compared to untreated cells, but were not as effective. The viability of yeast cells also decreased with longer freezing time. After 40 days of freezing, the viability of yeast cells decreased by 3 times across all conditions. The trends remained similar showing that PEF pretreated yeast cells were more resistant to freeze-induced injuries. The viability of cells without any pretreatment (no soaking in trehalose and no exposure to PEF) decreased to 1.4% ± 1.1%. Pretreatment by soaking improved viability up to 3% ± 0.3%. The highest viability (9.9% ± 2.1%) was achieved by yeast cells, which were exposed electric field pulse (E = 4 kV/cm) and soaked in the same solution with trehalose for 30 min prior to freezing.

**Figure 3:**
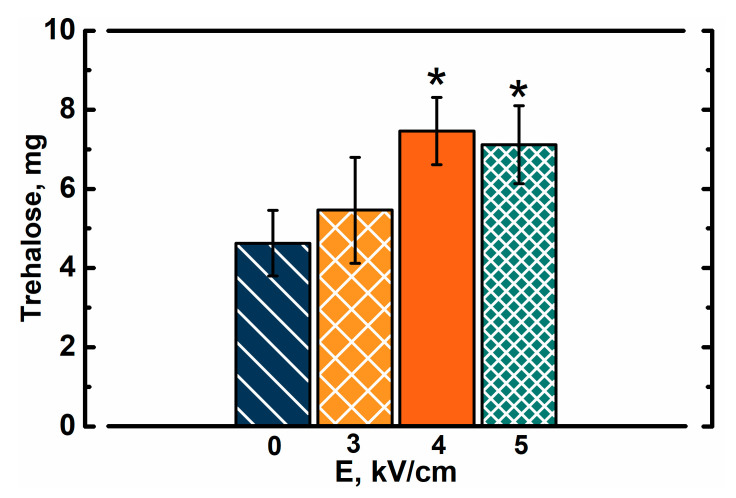
Amount of trehalose in 1 mg of dried yeast cells, which were soaked in trehalose solution for 30 min after PEF pretreatment. The drying procedure was performed after extraction of trehalose. Asterisks (*) indicate significant difference (*p* < 0.05) in trehalose concentration when compared to the PEF-untreated cells.

In order to validate whether PEF treatment improved the introduction of trehalose into yeast cells, we evaluated its concentration in yeast cells (µg of trehalose/mg of dry yeast mass). Extracts from yeast cell lysates were analyzed via chromatography (Figure 3).

Results show that within the same soaking time, the PEF pretreated cells absorbed more trehalose, which could theoretically be used to resist freeze-induced damage or even as a metabolite. Trehalose concentration increased with a rise in electric field strength. In PEF treated cells, the concentration of trehalose was up to 60% higher when compared to cells, which were only soaked in trehalose for 30 min. Interestingly, the soaking itself proved to significantly improve the content of intracellular trehalose (up to 4.6 ± 0.8 µg/mg) when compared to yeast cells soaked in sorbitol (0.06 ± 0.02 µg/mg). It is very likely that concentrations of intracellular trehalose in frozen samples were even higher before extraction. Preparation of yeast cell for extraction involved washing with sorbitol solution, which could induce some stress due to a change in composition of solution and leakage. This is especially important in the case of PEF-treated cells where the loss of intracellular compounds may be even higher due to the permeability of membranes. This experiment also shows that yeast cells in the exponential growth phase contain only minimal amounts of trehalose, which does not change within 45 min of soaking in sorbitol solution on ice. It was previously shown by Nakamura et al. that yeast cells in the exponential growth phase accumulate very small amounts of trehalose (<0.5 mg/g dry weight cell), while the amount of trehalose in mutant cells with ∆nth1 was higher (2.56 ± 0.15 mg/g dry weight cell) [30]. The same study showed that mutant cells with higher concentration of intracellular trehalose were around twice as resistant to freezing than wild type cells [30]. In our study the amount of trehalose in yeast cells (both PEF-treated and untreated) was of the same order as in ∆nth1 deficient cells.

Non-permeating cryoprotectants such as trehalose [10] were previously used for successful freezing procedures of yeast cells. There are several reasons why such compounds positively affect the viability of cells. First of all, they often result in a change of osmotic environment and subsequent partial removal of intracellular water, which in turn lowers the probability of intracellular ice crystal formation. Furthermore, it is likely that trehalose could contribute to the protection of membranes structural integrity, maintenance of phase separation, thus preventing leakage, and inhibiting fusion with surrounding membranes [31].

One of prospective applications where such a pretreatment could be of use is frozen dough. It was previously shown that the addition of trehalose can improve dough behavior under freezing conditions in terms of bread volume and texture characteristics [32]. Besides its cryoprotective functions, trehalose could be used by yeast cells as a carbon source during pre-fermentation process or after thawing. Yeast viability may affect gassing power, fermentation time and loaf volume, which is the determinant of consumers’ acceptability. Since pulsed electric fields can be employed as a method to improve freezing tolerance in yeast cell suspension, we believe that such a pretreatment of yeast cells could improve dough behavior under freezing conditions as well.

## 4. Conclusions

We showed that the PEF pretreatment could improve the freezing tolerance of yeast cells thus resulting in higher viability of up to 3 times. Such a pretreatment in trehalose solution resulted in higher concentration of intracellular trehalose (by up to 60%) when compared to PEF untreated cells. The optimal electric field strength in the case of single square shaped pulse with duration of 150 µs was 4 kV/cm. Such parameters also correspond to the threshold above which lethal effects of PEF treatment come into play. Soaking time had an effect on the viability after thawing only for PEF-treated cells. The optimal soaking time after PEF treatment in trehalose solution was 30 min. Investigation of intracellular trehalose contents confirmed that its concentration increased with rise in strength of the electric field.

The interest in finding a procedure, which can be used to modify yeast cells without genetic manipulations, has prompted us to invest effort in tailoring electroporation procedure for the introduction of cryoprotectant. On the basis of the data presented in this study we assumed that PEF pretreatment could enhance yeast behavior in dough after thawing by increasing the number of viable cells and subsequently improving their fermentation ability.

## Figures and Tables

**Table 1:** **Limit of detection** (LOD), limit of quantitation (LOQ), linearity range and retention time (RT) of trehalose.

Target ion, m/z	387
Ion extraction window (m/z)	386.5–387.5
RT, min	6.36
LOD, µg/mL	0.1
LOQ, µg/mL	0.4
Linearity range, µg/mL	0.1–50.0
Linear regression coefficients	0.9966

## Appendix A

**Table A1:** MS conditions used to monitor trehalose for the LC–MS based assay.

Parameter	Condition Used
Fragmentor Voltage	125 V
Skimmer Voltage	65 V
Capillary Voltage	3.0 kV
Source Gas Temperature	325 °C
Collision Gas	Nitrogen
Gas Pressure	35 psi
Gas Flow	10 L/min
